# Minimally Invasive Thoracic-Lumbar Stabilization Surgery for Infected Charcot Spine Arthropathy (ICSA) After Spinal Cord Injury

**DOI:** 10.7759/cureus.55039

**Published:** 2024-02-27

**Authors:** Fumihiro Arizumi, Keishi Maruo, Kazuya Kishima, Norichika Yoshie, Toshiya Tachibana

**Affiliations:** 1 Department of Orthopaedic Surgery / Spine Surgery, Hyogo Medical University, Nishinomiya, JPN

**Keywords:** 3d rod bending system, penetrating endplate screw, lateral interbody fusion, minimally invasive surgery, spinal cord injury, infected charcot spine arthropathy

## Abstract

Charcot spinal arthropathy (CSA) is a very rare condition that causes destruction and deformity of the spine due to impaired sensation. We report a case of an infected Charcot spine arthropathy (ICSA) treatment with spinal reconstruction surgery using a minimally invasive surgery (MIS) technique. A 49-year-old man who had a spinal cord injury (SCI) at age 19 presented with a destructive lesion in the L2/3 and a fistula in his lower back. Spinal reconstruction surgery using a penetrating endplate screw, lateral lumbar interbody fusion (LLIF), and a computer-assisted rod bending system were performed. A CT scan taken six months after surgery showed bony fusion. Reconstruction of the destructive spine is necessary to control the infection and symptoms due to kyphotic deformity for ICSA. Although the treatment of ICSA generally requires a highly invasive approach, we have achieved good clinical results with minimally invasive reconstructive surgery.

## Introduction

Charcot spine arthropathy (CSA) is a condition of severe destruction and instability of the spine due to lack of or diminished nociperception and proprioception [1,2]. Repeated microtrauma over many years causes destruction of the cartilage and intraarticular ligaments, and a narrowing of the intervertebral disc space [3,4]. CSA is commonly reported in case reports and small case series, therefore, it is difficult to determine the incidence or prevalence of CSA. Patients with CSA have a variety of clinical symptoms, many of which are nonspecific. The common symptoms include back pain, instability of the spine, spinal kyphotic deformity in the sitting position, and audible noises such as clicking from the spine while moving [5,6]. CSA was initially described as a complication of tertiary syphilis infection [7], but more recently has been reported after traumatic spinal cord injury (SCI) [8-10]. CSA patients after SCI may develop skin fistulas and urinary tract infections, which can lead to infection in destructive intervertebral space. There are few reports of infected Charcot spine arthropathy (ICSA) [11,12]. In addition, surgery is highly invasive because of the high degree of destruction involved [13]. To our knowledge, minimally invasive surgery (MIS) for ICSA has been never reported. We report a minimally invasive fixation of the ICSA after SCI.

## Case presentation

**Figure 1:**
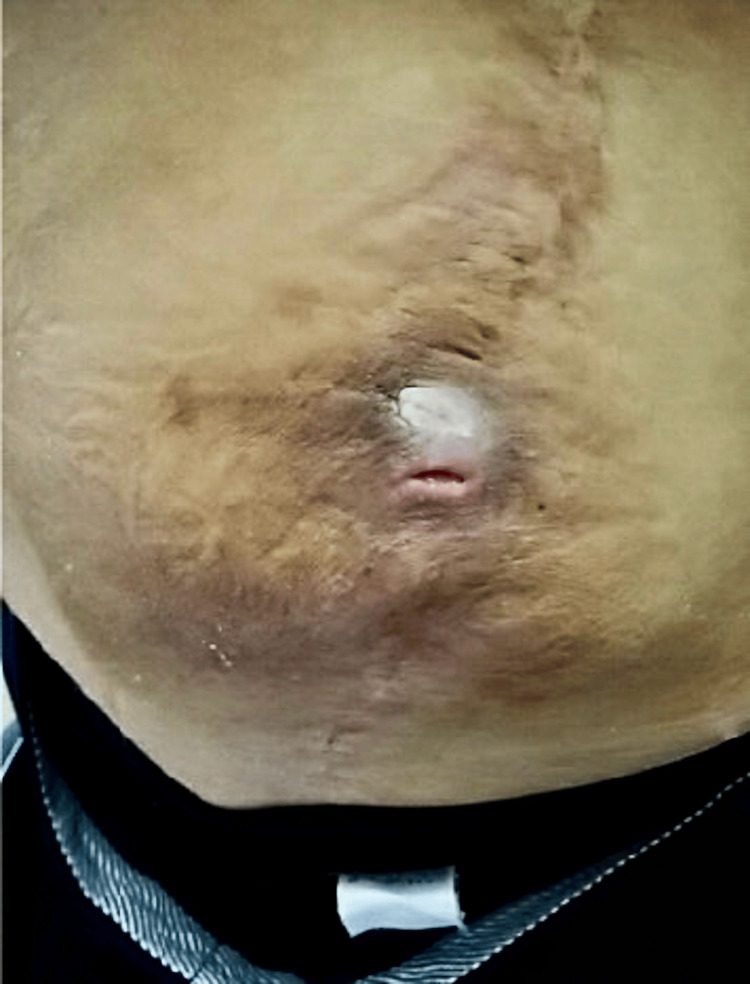
Kyphotic deformity and effusion of serous fluid from the fistula were observed in the patient’s lower back.

A 49-year-old man suffered an L2 dislocation fracture due to a motorcycle traffic accident and underwent T12 to L5 posterior decompression and fusion surgery in our hospital at age 19. Nevertheless, Frankel B's level of paralysis sequelae remained. Removal surgery was performed at five years postoperatively. He was aware of a mass on his lower back. An excisional biopsy was performed by the plastic surgeon. However, three months after surgery, the wound did not heal. He was referred to our hospital for back pain, appetite loss, and repeated fevers. A physical examination during the patient's first visit to our hospital revealed abnormal anterior lumbar bending and effusion of serous fluid from the fistula in his lower back (Figure 1). A bacteriologic study of the fistula showed methicillin-sensitive Staphylococcus aureus. Laboratory evaluation revealed a white blood cell count of 4870/μL, a C-reactive protein (CRP) level of 14.9 mg/dL, and a negative test for syphilis and tuberculosis.

**Figure 2:**
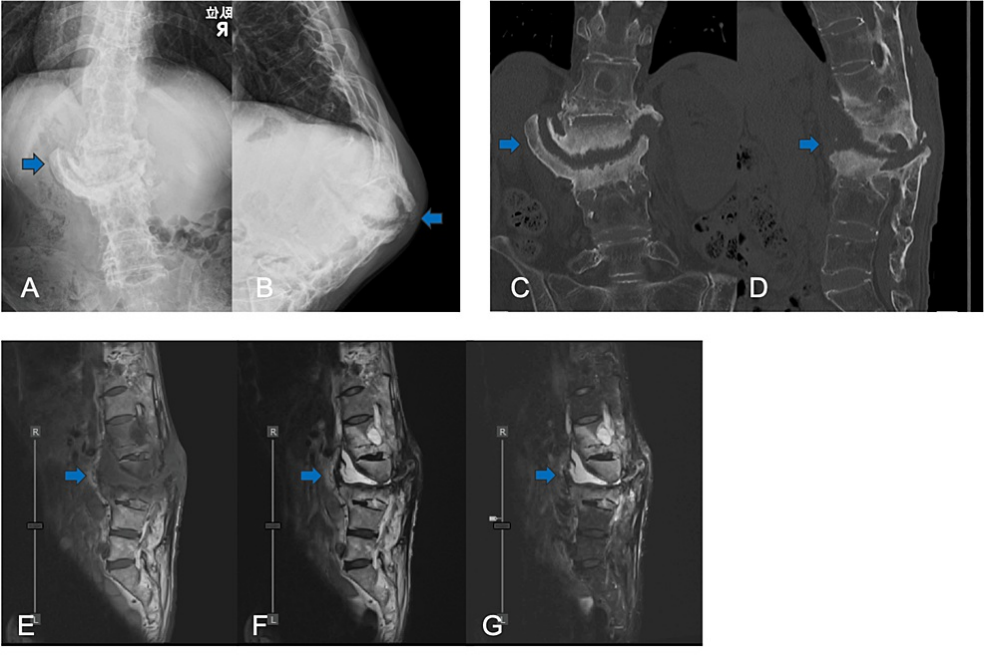
(A, B) Radiograph in the sitting position showing scoliosis, kyphosis, and spinal instability (blue arrows). (C, D) CT showing destructive change and large osteophyte on L2/3 (blue arrows). MRI sagittal T1-weighted (E), T2-weighted (F), and STIR (G) images showing continuous fluid from L2/3 to the posterior fistula (blue arrows).

X-ray taken from the seated position showed significant spinal scoliotic and kyphotic deformity (Figures 2A-2B). CT scans showed a fusion mass formation except at the L2/3 level, osteolysis mainly in the anterior L2 on the sagittal view, and large osteophyte on the coronal view (Figures 2C-2D). MRI demonstrated a low-intensity area in the T1-weighted image and a high-intensity area in the T2-weighted image from between the L2 and L3 vertebral bodies to the posterior fistula (Figures 2E-2G). After three months of oral antibiotic treatment (cefaclor), laboratory evaluation revealed a white cell count of 3430/μL and a CRP of 0.08mg/dL.

**Figure 3:**
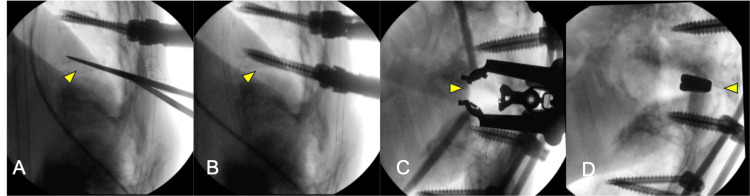
Intraoperative fluoroscopy. (A, B) Posterior fixation with PPS using penetrating endplate screw technique (yellow arrows). (C, D) Anterior reconstruction using the bioactive porous titanium spacer (yellow arrows). PPS: percutaneous pedicle screw

Anterior and posterior combined thoracic-lumbar stabilization surgery was performed. First, T11-L5 posterior fixation using percutaneous pedicle screws (PPS) was performed in the supine position. Because the bone quality was assumed to be fragile, to strengthen the screw, PPS was inserted by penetrating the endplate (T11, 12 were double; L4 and L5 were single) (Figures 3A-3B). A titanium alloy rod with a diameter of 5.5mm was used. Rod bending was performed using the computer-assisted rod bending system (Bendini, NuVasive, Inc., San Diego, CA) to avoid pulling out of percutaneous pedicle screws. After rod bending, rods were inserted under the fascia and fastened with set screws. Next, the right 11th rib was resected approximately 4 cm and entered into the posterior pararenal space via a diaphragmatic approach in the left lateral decubitus position. A retractor (MaXcess® 4, NuVasive, Inc.) was placed between the L2 and L3 vertebral bodies, and intervertebral curettage and irrigation were performed. The bioactive porous titanium spacer (XTAL; Nuvasive Japan, Tokyo, Japan, and Osaka Yakin Kogyo Co. Ltd., Osaka, Japan) was inserted between the L2 and L3 vertebral bodies (Figures 3C-3D).

**Figure 4:**
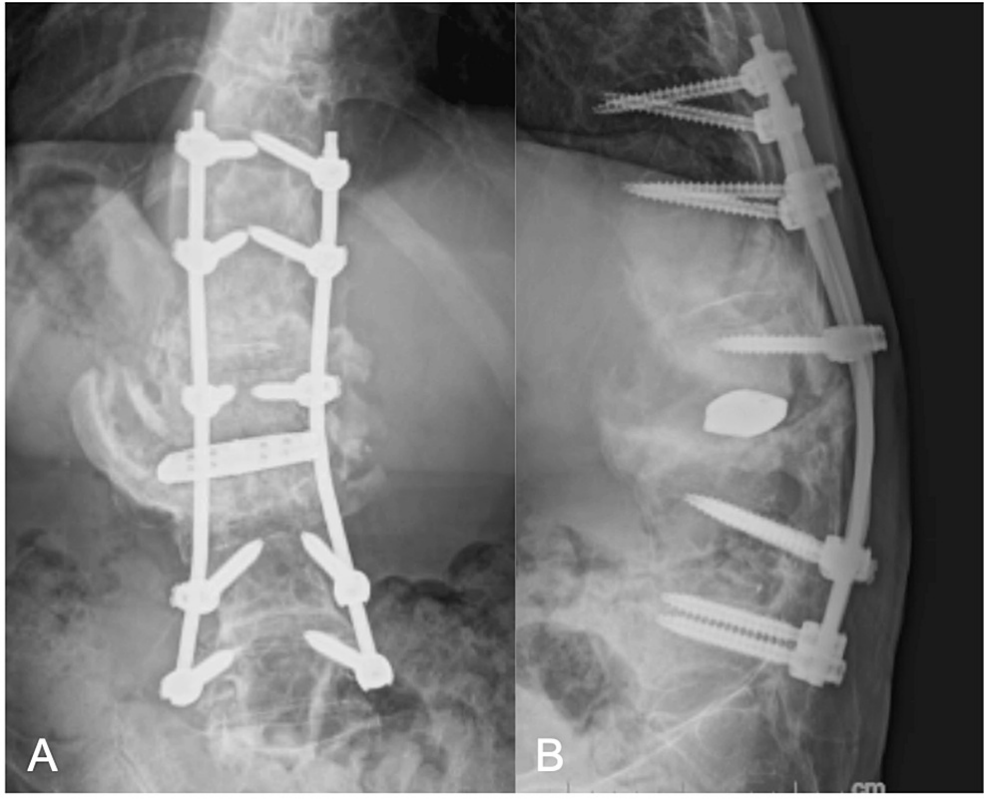
A, B. Postoperative radiograph showing anterior reconstruction with LLIF and posterior spinal fixation with PPS. LLIF: lateral lumbar interbody fusion; PPS: percutaneous pedicle screw

No bacteria were detected in the tissue collected at the time of surgery. The operative time was 144 minutes for the posterior approach and 95 minutes for the anterior approach, and the intraoperative blood loss was 270 ml. After surgery, the fever and back pain disappeared, and the appetite improved. The local kyphosis angle at T11 to L5 improved from 75° (pre-op) to 19 °(post-op) (Figures 4A-4B).

**Figure 5:**
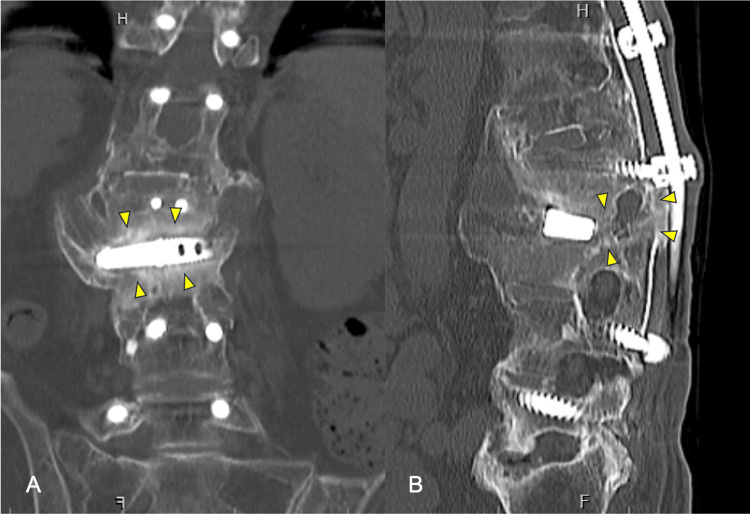
CT coronal (A) and sagittal (B) images showing bony fusion between L2 and L3 vertebral bodies at the six-month postoperative visit (yellow arrows).

CT at six months postoperatively showed bony fusion between L2 and L3 vertebral bodies (Figure 5).

## Discussion

The mechanism of CSA may be excessive loading of the thoracolumbar spine by transferring movements due to diminished nociperception and proprioception [1,2,14]. ICSA after SCI is commonly caused by a skin fistula or infected pressure ulcer, although some causes are hematogenous [9,15]. In this case, a bony fusion between L2/3 was not achieved despite the stabilization surgery for the L2 dislocation fracture. It is considered that repeated excessive loading and microtrauma between the L2/3 over many years caused high instability and kyphotic deformity of the spine. The severe kyphotic deformity of the spine resulted in skin fragilization and mass formation, which led to a skin fistula triggered by plastic surgery.

Conservative treatment is not expected to control inflammation or improve symptoms, and spinal reconstruction is recommended for ICSA [16]. It is important to ensure a stable surgical environment to stop the destructive processes and control the infection. In our case, the CRP level became negative after three months of antibiotic therapy, but back pain and anorexia remained unchanged. Because of the expected exacerbation of inflammation due to residual spinal instability, surgery was performed for ICSA. Since rigid fixation is necessary for the reconstruction of the spine, a combination of anterior and posterior fixation has been reported in many cases [2]. MIS is desirable, but the high degree of spinal instability requires a highly invasive approach. There have been some reports of revisions due to the loosening of screws and postoperative infections [16]. Microtrauma caused by ICSA also results in a bad skin condition and is more likely to cause postoperative wound infections. MIS is desirable to prevent the occurrence of postoperative infection. Therefore, rigid and minimally invasive spinal reconstruction is necessary for ICSA.

In general, iliac bone grafts, fibula bone grafts, and titanium mesh cages are used in anterior reconstruction for ICSA [11-13]. We selected a bioactive porous titanium spacer for lateral lumbar interbody fusion (LLIF) with stronger intervertebral stability for anterior column reconstruction in this case. The advantage of this spacer is that it does not require autogenous bone grafting. Fujibayashi et al. reported that the bone fusion rate in single- and double-level LLIF using bioactive porous titanium spacers was comparable to that of conventional hollow cages with autogenous bone grafts [17]. In our case, bone fusion was achieved at six months postoperatively without an autogenous bone graft.

Insertion of a screw into an osteoporotic vertebra is associated with the risk of screw pull-out. We used two MIS techniques to prevent screw pull-out with bone fragility in this case. The first of these is the penetrating endplate screw technique using PPS. The screw was inserted upward from the pedicle and penetrated the upper endplate of the vertebral body and the lower endplate of the adjacent cranial vertebral body. Takeuchi et al. reported that single or double endplate penetrating screws (SEPS/DEPS) for diffuse idiopathic skeletal hyperostosis (DISH) provided stronger fixation than the conventional PPS technique [18]. The second is a computer-assisted rod bending technique. After registering the screw head using a digitizer and an infrared motion tracking camera, the proprietary rod bender was used to form the rod. This 3D rod bending system allows the rod to be inserted without stressing the screw [19]. In this case, the patient's sitting balance in the wheelchair was stabilized in a slightly kyphotic position, so excessive kyphosis correction was not performed.

## Conclusions

Local spinal stabilization is necessary for ICSA with persistent inflammation. However, in general, surgery for ICSA is often highly invasive. In this case, MIS using LLIF and PPS resulted in controlling the inflammation, leading to symptomatic improvement. The combination of various MIS techniques provides a strong spinal fusion force and is an option for the treatment of ICSA.
